# The effect of dietary fiber supplement on prevention of gestational diabetes mellitus in women with pre-pregnancy overweight/obesity: A randomized controlled trial

**DOI:** 10.3389/fphar.2022.922015

**Published:** 2022-08-29

**Authors:** Dong-Yao Zhang, De-Cui Cheng, Yan-Nan Cao, Yao Su, Li Chen, Wen-Yu Liu, Yue-Xin Yu, Xian-Ming Xu

**Affiliations:** Department of Obstetrics and Gynecology, Shanghai General Hospital, Shanghai Jiao Tong University School of Medicine, Shanghai, China

**Keywords:** gestational diabetes mellitus, dietary fiber, overweight/obesity, prevention, pregnancy, prospective study

## Abstract

**Objective:** To investigate the effect of dietary fiber intake during pregnancy on the prevention of gestational diabetes mellitus (GDM) in women who are overweight/obese prior to pregnancy.

**Methods:** This randomized controlled trial was conducted in Shanghai General Hospital from June 2021 to March 2022. A total of 98 women who reported BMI≥24 kg/m^2^ prior to pregnancy were recruited before their 20th gestational week, and randomly (simple random allocation) assigned to the fiber supplement group (12 g of dietary fiber power twice daily) and the control group (standard prenatal care) from 20 to 24^+6^ gestational weeks. Both groups received nutrition education and dietary advice during the study. GDM diagnosis was performed by an oral glucose tolerance test (OGTT) at 25–28 weeks’ gestation. Data are presented as means with SD, as medians with IQR, or as counts with percentages as appropriate. Comparisons were conducted using a *t*-test, Mann-Whitney *U* test, and χ^2^ test, respectively.

**Results:** The incidence of GDM was significantly reduced in the fiber supplement group compared with the control group: 8.3 vs. 24.0% (χ^2^ = 4.40, *p* = 0.036). At OGTT, the mean fasting plasma glucose in the fiber supplement group was significantly lower than before the intervention (4.57 ± 0.38 mmol/L vs. 4.41 ± 0.29 mmol/L, *p* < 0.01) but not in the control group (4.48 ± 0.42 mmol/L vs. 4.37 ± 0.58 mmol/L, *p* = 0.150). Compared with the control group, the TG and TG/HDL-C ratio levels in the intervention group were significantly higher than those in the control group (2.19 ± 0.54 mmol/L vs. 2.70 ± 0.82 mmol/L and 1.19 ± 0.49 vs.1.63 ± 0.63, respectively, all *P*＜0.05). The body weight gain was significantly lower in the fiber supplement group than the control group (1.99 ± 1.09 kg vs. 2.53 ± 1.20kg, *p* = 0.022). None of the women randomized to the fiber supplement group experienced preterm birth (<37 weeks gestation) compared with 12.0% in the control group (*p* = 0.040). Excessive weight gain (total weight gain >11.5 kg for overweight, and >9.0 kg for obesity) occurred in 46.7% of women in the fiber supplement group compared with 68.0% in the control group (*p* = 0.035). There were no differences in other maternal and neonatal outcomes.

**Conclusion:** Increased dietary fiber intake in pregnant women who were overweight/obese prior to pregnancy may reduce the risk of GDM, excessive weight gain, and preterm birth, but it did not improve blood lipids.

## Introduction

Gestational diabetes mellitus (GDM) is defined as any glucose intolerance firstly recognized or onset in pregnancy. Considered one of the most common metabolic diseases experienced by women during pregnancy, GDM is associated with an increased risk of adverse maternal and neonatal outcomes, including macrosomia, neonatal hypertensive disorders, cesarean delivery, and an increased risk of developing type 2 diabetes later in life for the mother (McIntyre et al., 2019; Chen et al., 2021). It is estimated that approximately 14% of pregnancies are affected by GDM worldwide, and the prevalence is increasing as rates of overweight/obesity continue to rise among women of childbearing age (Stern et al., 2021).

Women with pre-pregnancy overweight/obesity (defined using ethnic-specific thresholds of BMI ≥24 kg/m^2^ for Chinese, and ≥25 kg/m^2^ for White, Black, and mixed), a higher risk factor for GDM, are associated with increased levels of inflammatory markers, which contribute directly to the development of insulin resistance and GDM(Yen et al., 2019; Godfrey et al., 2021). Chu and others reported ORs of developing GDM were 2.14 (95% CI: 1.82–2.53), 3.56 (95% CI: 3.05–4.21), and 8.56 (95% CI: 5.07–16.04) among overweight (BMI ≥25 kg/m^2^), obesity (BMI ≥30 kg/m^2^), and severely obesity (BMI ≥40 kg/m^2^), respectively, compared with normal-weight women (Chu et al., 2007). Lifestyle interventions involving exercise and a healthy diet were strongly recommended owing to medical treatments that pose potential threats to the fetus (Juan and Yang, 2020; Li et al., 2021). However, the DALI (Vitamin D and lifestyle intervention for GDM prevention) study group reported that among pregnant women with obesity, healthy eating and physical activity alone were unlikely to prevent GDM development, nor was it a cost-effective early intervention to decrease fasting glucose and insulin sensitivity (Simmons et al., 2017), emphasizing the need for new preventive approaches.

Dietary fiber consists of nondigestible carbohydrates and lignin, which are not digested and absorbed by the human body (Barber et al., 2020). Several studies show that increasing the intake of dietary fiber during pregnancy benefits a lot of women by reducing excessive weight gain, insulin resistance, and lowering the risk of glucose intolerance (Pretorius and Palmer, 2020; Jaworsky et al., 2021; Zhang et al., 2021). Fruits and vegetables, along with whole-grains, are excellent sources of dietary fiber, and the Food and Drug Administration (FDA) has also approved that foods high in fruits, vegetables, and whole-grains have multiple health benefits for pregnant women (Zerfu and Mekuria, 2019). The Chinese Dietary Reference Intakes (DRIs) 2013 recommends a minimum of 25 g/day of dietary fiber during pregnancy, while the average total dietary fiber intake in Chinese pregnant women (14.9 g/day) is far below the recommended daily intake (Liu F.-L. et al., 2015; Liu F. L. et al., 2015; Bailey et al., 2019; Zerfu and Mekuria, 2019; Chen et al., 2020; Roskjaer et al., 2021). It was based on the fact that a larger proportion of dietary fiber intake is essential to reduce the chance of developing metabolic complications during pregnancy. Therefore, we designed and conducted a randomized controlled trial in pre-pregnancy overweight/obese women to investigate whether intervention with dietary fiber supplement, compared with standard prenatal care, taken during pregnancy, would reduce the risk of GDM and improve maternal pregnancy outcomes.

## Materials and methods

### Design overview

We conducted a unicentric, clinic-based, randomized controlled trial between June 2021 and March 2022 at the Department of Obstetrics and Gynecology at the Shanghai General Hospital, Shanghai Jiao Tong University School of Medicine, Shanghai, China. This study was approved by the Shanghai General Hospital of School of Medicine, Shanghai Jiao Tong University ethics committee (2020KY098) and was registered on Chinese Clinical Trial Registry website (ChiCTR2000036575).

### Participants

Sample size was calculated based on a 66.7% reduction in GDM frequency with the use of dietary fiber supplement (from 36 to 12%), with a statistical power of 80% (*α* = 0.05), as well as allowing for 10% attrition, called for the recruitment of 102 women (92 completers) (Yan et al., 2019). In the end, a total of 104 pregnant women were recruited in the Shanghai General Hospital, and 98 of them completed the study. Simple randomization was applied with an allocation of 1:1 using Microsoft Excel 2019 random number generator in each group.

The inclusion criteria were as follows: 1) self-reported pre-pregnancy BMI ≥24 kg/m^2^(Wang et al., 2017); 2) ＜20 weeks of gestation; 3) singleton pregnancy; 4) not having diabetes mellitus; 5) do not current use of medications that might affect glucose metabolism (metformin, glucocorticoids, immunosuppressants, antipsychotics); 6) willingness or ability to provide written informed consent.

Participants in this study would be removed if one of the following occurred: 1) women request to be removed from the study or withdraw consent for any reason; 2) adverse events that made it difficult to continue; 3) compliance with intervention less than 50% of the time; or 4) women who lost contact during the follow-up.

### Intervention

The intervention was from 20 to 24^+6^ weeks of gestation, and all participants received standard prenatal care, including nutrition education and dietary advice by nutritionists based on the Chinese Dietary Guidelines for Pregnant Women (Wang et al., 2016). The guidelines recommend: 1) take iron-rich foods (20–50 g red meat/day), and iodized salt; 2) increase intake of milk (500 g/day), and the amounts of fish, poultry, eggs and lean meat increased by 50 g/day; 3) total carbohydrate daily intake≥130 g; 4) moderate physical activity (≥30 min/day) to maintain recommended gestational weight gain (overweight: 7–11.5 kg, and obesity: 5–9 kg). 5) smoking cessation and keep good spirits. In addition to the measures above mentioned, the fiber supplement group was given 1 bag (12 g) of soluble dietary fiber powder (Nutrasumma, Qingdao Nutrasumma Health Technology Co., Ltd.) twice daily, which contained 51.93 of kcal energy, 3.31 g of carbohydrates, and 9.78 g of dietary fiber. Furthermore, participants in both groups also completed food frequency questionnaires (FFQs) to assess dietary intake pre and post intervention. The doctors were responsible for timely management of any adverse reaction during the study. All pregnant women enrolled in the study underwent a 75 g oral glucose tolerance test (OGTT) at 25–28 weeks of gestation.

Flowchart 1. Enrollment of participants in the study.

### Outcomes and data collection

Outcomes: The primary outcome measure was the incidence of GDM. Secondary outcomes included the leaves of blood glucose and lipids, diet changes, gestational weight gain, and maternal and neonatal outcomes.

Anthropometric measures: Pre-pregnancy BMI (kg/m^2^) was calculated using the self-reported data from participants. Body weight (kg) was measured while the participants were wearing light clothing at a gestational age of 20 and 25 weeks, and weight gain (kg) was evaluated at 25 weeks of gestation. Blood pressure (mmHg) was measured at the first two visits with an adequate armlet when the participants had been seated for at least 10 min, after which 3 blood pressure measurements were recorded at intervals of 10–15 min and the mean values were adopted. Data on maternal and neonatal outcomes were abstracted from the electronic medical record.

Biochemical variables: Blood samples were drawn between 07.30 and 09.00 a.m., after an overnight fast. Blood samples were collected and sent to be analyzed within 3 h. A biochemical autoanalyzer (ADVIA2400 Chemistry System, Siemens Healthcare Diagnostics Ltd., Germany) was adopted to analyze the plasma glucose (fasting plasma glucose [FPG], 1-h postprandial plasma glucose [1 hPG], 2-h postprandial plasma glucose [2 hPG]) and blood lipid profiles, including triglycerides (TG), total cholesterol (TC), high-density lipoprotein cholesterol (HDL-C), and low-density lipoprotein cholesterol (LDL-C). Serum glycated hemoglobin (HbA1c) was measured by high-pressure liquid chromatography using HLC-723G8 instruments (Tosoh, Tokyo, Japan). These biomarkers were mainly measured at baseline and at 25–28 weeks of gestation.

Diet evaluation: Energy and dietary fiber intakes were calculated from online FFQs (Zhang et al., 2015), including a total of 39 items from nine food groups, which are cereals (rice, pasta, roughage, bread, and crackers), vegetables (roots, stem, fruit, and leaf vegetables), fruits (fresh, canned, dried), meats and eggs (pork, beef, mutton, poultry, fish, and eggs), beans (soya-bean milk and other soy products.), nuts, milk and milk products (milk, yoghurt, and cheese), oil (liquid oil), and beverages (fresh fruit juice, juice drinks, carbonate drinks, and coffee). Participants were required to recall their usual frequency and portion size of consuming each food item in the past 5 weeks at 20 and 25 weeks of gestation. Food intake frequency was measured as per day, per week, per month, or never. Food models representing standard portion sizes of relevant food items were presented to participants to help them estimate their usual consumption. Subsequently, the mean daily total energy intake (including carbohydrate, protein, fat, and energy intake) and dietary fiber were evaluated using the Chinese Food Composition Table (Yang et al., 2009). Trained dietary interviewers helped all participants complete the FFQ, ensuring the accuracy of the data collected.

### GDM diagnosis

The diagnosis of GDM was based on a 75 g oral glucose tolerance test (OGTT) at 25–28 weeks of gestation, according to the International Association of Diabetes and Pregnancy Study Groups (IADPSG) criteria (Weinert, 2010). Diagnosis of GDM was confirmed if one or more values were at or above the threshold level: fasting plasma glucose (FPG)≥5.1 mmo1/L; OGTT 1 h plasma glucose≥10.0 mmol/L; OGTT 2 h plasma glucose≥8.5 mmol/L.

### Statistical analysis

Normal distribution of data was evaluated using the Shapiro-Wilk test. Continuous variables were shown as means with SD for normally distributed data, or as medians with IQR for non-normally distributed data, and categorical variables are presented as counts with percentages. The *t*-test was used to compare continuous variables with normal distribution, the Mann-Whitney *U* test was used to compare continuous variables with non-normal distribution, and χ two test or Fisher’s exact test for categorical variables, when applicable. Statistical significance was defined as a *p* value < 0.05. Statistical analyses were performed using SPSS statistical software (IBM, version 25.0 for Windows).

## Results

### Baseline characteristics

In the end, a total of 52 women were randomized to the fiber supplement and 52 to the control group, and 48 and 50 completed the follow-up, respectively. (Flowchart 1). Demographic and clinical characteristics did not significantly differ between the fiber supplement group and the control group at baseline (Table 1).

**TABLE 1:** Maternal demographic and medical characteristic in fiber supplement and control groups.

Characteristic	Control (*n* = 50)	Fiber supplement (*n* = 48)	*P*
Gestational age (weeks) at enrollment	12.30 (12.00–13.03)	12.5 (11.60–13.55)	0.636
Gestational age (weeks) at OGTT	25.30 (25.08–26.10)	25.45 (25.20–26.008)	0.369
Age (years)	29.96 ± 4.07	31.13 ± 4.21	0.167
BMI (kg/m^2^)	25.90 (24.54–28.35)	26.05 (24.89–28.05)	0.815
Systolic blood pressure, (mmHg)	116.60 ± 12.76	119.42 ± 11.93	0.262
Diastolic blood pressure, (mmHg)	71.68 ± 9.11	72.19 ± 10.03	0.794
First pregnancy, n (%)	20 (40)	11 (22.9)	0.069
Family history of diabetes, n (%)	8 (16)	12 (25)	0.269
PCOS, n (%)	3 (6)	7 (14.6)	0.285
Adverse pregnancy history, n (%)	10 (20)	6 (12.5)	0.315

Data are expressed as mean ± SD, median (IQR), or n (%); OGTT, oral glucose tolerance test; BMI, body mass index; PCOS, polycystic ovary syndrome.

**Figure:**
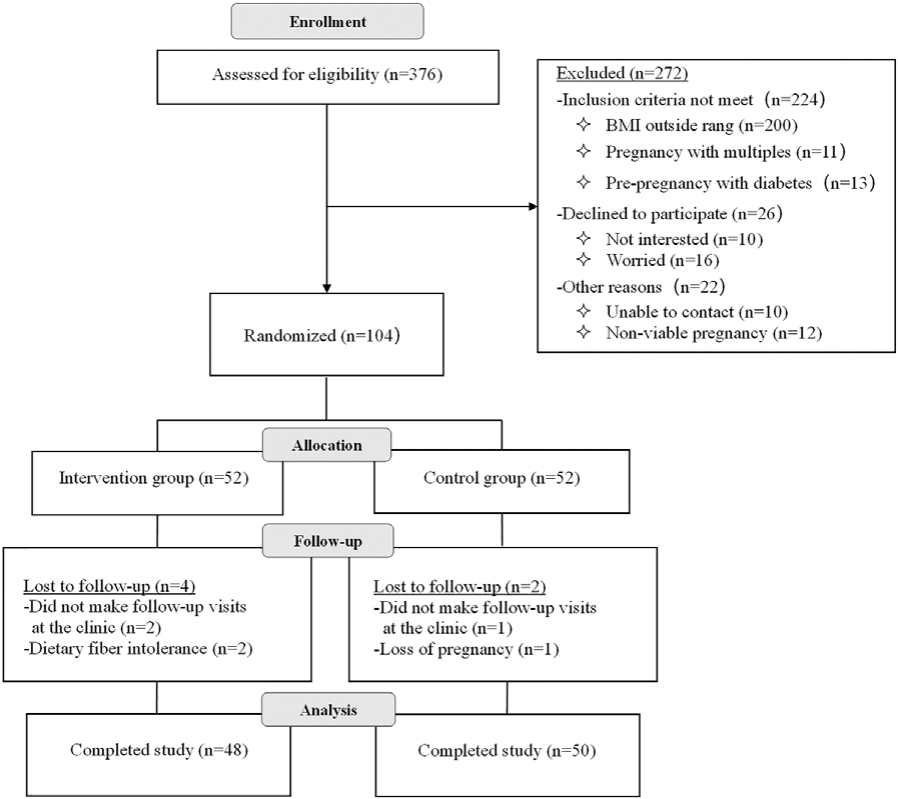
.

### Incidence of GDM

OGTT, scheduled for all participants, was performed at a mean of 25.57 ± 1.00 weeks of gestation. Among women in the control group, 12 of 50 (24.0%) were diagnosed with GDM according to IADPSG criteria, whereas only four of 48 (8.3%) were diagnosed with GDM in the fiber supplement group. We found a statistically significant difference in the incidence of GDM between the groups (χ^2^ = 4.400, *p* = 0.036) (Table 3).

### FPG and HbA1c

The comparison between the two groups showed no significant differences with the levels of FPG and HbA1c at the baseline and the end (all *p* > 0.05). After 5 weeks of intervention, HbA1c levels in both groups were slightly decreased, and FPG levels were only decreased slightly in the fiber supplement group compared to baseline values (all *p* < 0.05) (Table 2).

**TABLE 2:** Glucose and lipids levels in overweight/obese mothers in fiber supplement and control groups pre and post-intervention.

Variable	Control (*n* = 50)		*P*	Fiber supplement (*n* = 48)		*P*
	Baseline	Post intervention		Baseline	Post intervention	
HbA1c (%)	5.16 ± 0.37	5.06 ± 0.37	0.033	5.18 ± 031	5.00 ± 0.33	<0.001
FPG (mmol/L)	4.48 ± 0.42	4.37 ± 0.58	0.150	4.57 ± 0.38	4.41 ± 0.29	<0.001
TC (mmol/L)	4.76 ± 0.77	5.88 ± 0.81	<0.001	4.78 ± 0.96	5.82 ± 1.14	<0.001
TG (mmol/L)	1.51 ± 0.40	2.19 ± 0.54	<0.001	1.66 ± 0.61	2.70 ± 0.82*	<0.001
HDL-C (mmol/L)	1.44 ± 0.32	1.81 ± 0.31	<0.001	1.46 ± 0.36	1.74 ± 0.38	<0.001
LDL-C (mmol/L)	2.63 ± 0.63	3.19 ± 0.81	<0.001	2.64 ± 0.86	3.19 ± 1.11	<0.001
TG/HDL-C	1.11 ± 0.42	1.19 ± 0.49	<0.001	1.26 ± 0.45	1.63 ± 0.63*	0.001

Data are expressed as mean ± SD; Baseline, gestational age (weeks) at enrollment; * Significantly different from control (*p* < 0.001 for TG; *p* = 0.001 for TG/HDL-C); TG/HDL-C, triglycerides to high-density lipoprotein cholesterol ratio.

### Postprandial plasma glucose

OGTT measured between 25 and 28 weeks of gestation revealed that 1 hPG, 2 hPG, the difference of lhPG and FPG, and IAUC did not differ between the two groups after intervention (*p* > 0.05). However, the value decreased from 1 hPG to 2 hPG was significantly lower in the control group (0.91 ± 1.18 mmol/L) compared with the fiber supplement group (1.49 ± 1.54 mmol/L) (*p* = 0.037), and the value increased from FPG to 2 hPG in control group (2.47 ± 1.60 mmol/L) was significantly (*p* = 0.042) higher than the fiber supplement group (1.90 ± 1.07 mmol/L). The results are shown in Table 3.

**TABLE 3:** Comparison of OGTT between the two groups post intervention.

	Control (*n* = 50)	Fiber supplement (*n* = 48)	*P*
GDM	12 (24.0)	4 (8.0)	0.036
1 hPG (mmol/L)	7.74 ± 2.13	7.81 ± 1.19	0.850
2 hPG (mmol/L)	6.83 ± 1.83	6.31 ± 1.12	0.094
Difference of 1 hPG and FPG (mmol/L)	3.38 ± 1.92	3.39 ± 1.11	0.955
Difference of 1 hPG and 2 hPG (mmol/L)	0.91 ± 1.18	1.49 ± 1.54	0.037
Difference of 2 hPG and FPG (mmol/L)	2.47 ± 1.60	1.90 ± 1.07	0.042
IAUC	4.61 ± 2.60	4.34 ± 1.24	0.519

Data are expressed as mean ± SD or n (%); OGTT, oral glucose tolerance test; 1 hPG.1-h postprandial plasma glucose; 2 hPG, 2-h postprandial plasma glucose; FPG, fasting plasma glucose; IAUC: incremental area under curve.

### Blood lipids

At baseline, no significant difference was found in the levels of TG, TC, HDL-C, and LDL-C between the two groups (all *p* > 0.05). After intervention, TG, TC, HDL-C, LDL-C, and TG/HDL-C levels in both groups significantly increased with the progression of gestational age (all *p* ≤ 0.001). The levels of TG and TG/HDL-C ratio in the fiber supplement group were significantly higher than those in the control group (2.19 ± 0.54 mmol/L vs. 2.70 ± 0.82 mmol/L and 1.19 ± 0.49 vs.1.63 ± 0.63, respectively, all *p* < 0.05). As seen in Table 2.

### Diet Changes

Dietary intake data during the study are displayed in Table 4. The daily calorie, carbohydrate, protein, fat, and the proportion of daily calories from protein and fat intake were not differ significantly between groups before and after the intervention (all *p* > 0.05). The proportion of daily calories from carbohydrates and the median daily intakes of dietary fiber in the two groups were similar before the intervention but statistically different during the intervention period (57.36 ± 4.31 vs. 55.25 ± 5.56 and 15.25 vs. 33.81g, respectively, all *p* < 0.05). The mean increase of total calories in the control group (261.57 kcal) was significantly higher than the fiber supplement group (192.78 kcal); the mean intake of carbohydrates increased in the control group was significantly higher than the fiber supplement group (24.56 ± 74.10 g vs. 0.06 ± 29.52g, *p* = 0.034). In addition, participants randomized to the control group had a higher increase in daily protein intake than the fiber supplement group (15.76 ± 8.03 g vs. 10.92 ± 13.06g, *p* = 0.031), and there was no difference in fat.

**TABLE 4:** Comparison of dietary intake within and between the group at the base and post intervention.

Variable	Control (*n* = 50)	Fiber supplement (*n* = 48)	*P*
Calorie (kcal/day)			
Baseline	1580.92 (1375.43–1857.35)	1643.85 (1426.55–2010.49)	0.290
Post intervention	1892.26 ± 296.35	1886.91 ± 321.33	0.932
Energy from carbohydrates			
Baseline (%)	59.84 ± 6.88	61.15 ± 6.06	0.322
Post intervention (%)	57.36 ± 4.31	55.25 ± 5.56	0.039
Baseline (g/day)	247.48 ± 79.20	262.49 ± 68.59	0.319
Post intervention (g/day)	272.03 ± 51.64	262.55 ± 59.31	0.400
Energy from protein			
Baseline (%)	12.00 (11.00–13.00)	12.00 (11.00–13.00)	0.778
Post intervention (%)	14.00 (12.00–14.25)	13.00 (12.00–14.00)	0.376
Baseline (g/day)	46.57 (40.20–56.97)	48.54 (39.47–58.61)	0.522
25 weeks (g/day)	63.71 ± 10.49	61.8 ± 11.34	0.398
Energy from fat			
Baseline (%)	26.36 ± 5.80	25.25 ± 5.51	0.334
Post intervention (%)	27.52 ± 3.85	28.19 ± 4.41	0.426
Baseline (g/day)	43.98 (37.88–52.86)	46.03 (38.09–52.80)	0.704
Post intervention (g/day)	57.71 ± 11.11	58.44 ± 11.26	0.746
Energy intake changes§			
Calorie (kcal/day)	261.57 ± 173.69	192.78 ± 128.67	0.029
Carbohydrates (g)	24.56 ± 74.10	0.06 ± 29.52	0.034
Protein (g)	15.76 ± 8.03	10.92 ± 13.06	0.031
Fat (g)	11.24 ± 13.23	11.17 ± 14.32	0.979
Dietary fiber (g)			
Baseline	15.00 (12.63–18.94)	15.37 (12.18–18.05)	0.693
Post intervention	15.25 (13.25–18.19)	33.81 (31.81–36.06)	<0.001

Data are expressed as mean ± SD or median (IQR); §Energy intake changes pre and post intervention.

### Maternal weight control

There were no significant differences in body weight before and after intervention between the two groups (all *p* > 0.05). However, after the intervention, women in the intervention group (1.99 ± 1.09 kg) showed significantly lower weight gain than the control group (2.53 ± 1.20 kg), and the difference was statistically significant (*p* = 0.022). As shown in Table 5.

**TABLE 5:** Weight control and Outcomes of pregnancy complications, delivery events, and neonatal in fiber supplement and control groups.

Characteristic	Control (*n* = 50)	Fiber supplement (*n* = 48)	*P*
Weight			
W20 (kg)	72.20 ± 8.54	71.66 ± 6.97	0.733
W25 (kg)	74.73 ± 8.673	73.65 ± 6.92	0.498
W25 - 20 (kg)	2.53 ± 1.20	1.99 ± 1.09	0.022
Maternal			
Pregnancy-induced hypertension, n (%)	2 (4.0)	4 (8.3)	0.636
Preeclampsia, n (%)	3 (6.0)	4 (8.3)	0.955
Polyhydramnios, n (%)	0 (0.0)	1 (2.1)	0.490
Premature rupture of membranes, n (%)	7 (14.0)	7 (14.6)	0.934
Postpartum hemorrhageǁ, n (%)	1 (2.0)	2 (4.2)	0.971
Excessive weight gain※, n (%)	34 (68.0)	21 (46.7)	0.035
Inadequate weight gain※, n (%)	3 (6.0)	7 (15.6)	0.238
Weight gain per week from baseline to delivery (kg/week)	0.32 ± 0.13	0.29 ± 0.13	0.162
Cesarean, n (%)	31 (62.0)	37 (77.1)	0.105
Neonatal			
Gestational age at delivery (weeks)	39.20 (38.45–40.20)	39.10 (38.33–40.20)	0.864
Preterm (<37 weeks), n (%)	6 (12.0)	0 (0.0)	0.040
Birth weight (g)	3325.00 (3077.50–3702.50)	3355.00 (3100.00–3637.50)	0.884
Macrosomia (≥4,000 g), n (%)	5 (10.0)	3 (6.3)	0.758
Small for gestational age (<2,500 g), n (%)	4 (8.0)	2 (4.2)	0.712

Data are expressed as mean ± SD, median (IQR), or n (%); W20, maternal weight at 20 weeks; W25, maternal weight at 25 weeks; W25-20, maternal weight increased between 20 and 25 weeks; ǁ Vaginal birth ≥500 ml, cesarean birth ≥1000 ml; ※Excessive weight gain, overweight (BMI≥24 kg/m^2^) > 11.5 kg, obesity (BMI≥28 kg/m^2^) > 9.0 kg; Inadequate weight gain: overweight (BMI≥24 kg/m^2^) < 7.0 kg, obesity (BMI≥28 kg/m^2^) < 5.0 kg.

### Maternal and neonatal outcomes

Maternal and neonatal outcomes are presented in Table 5. In women taking dietary fiber, none experienced premature birth compared with 12.0% in the control group (*p* = 0.040), and 46.7% gained more than the recommended amount, which was significantly lower than 68.0% in the standard prenatal care group (*p* = 0.035). There were no other statistically significant differences in any maternal and neonatal outcome measures between the two groups.

### Adherence and adverse effects

Adherence to the intervention was good overall. 89.58% (43/48) taking 85% or more and 10.42% (5/48) between 50 and 80% of the provided dietary fiber powder. The compliance calculated from the returned dietary fiber powder indicated that a mean of 94.70% of the powder had been consumed.

Adverse effects of the dietary fiber powder were reported by five of 52 (9.62%) of the women; three of those reported some degree of diarrhea or bloating in the early 3–5 days of eating, and the other two women voluntary withdrew from the study due to mild to moderate abdominal pain.

## Discussion

The aim of the study was to evaluate the effect of dietary fiber supplement on prevention of GDM and improving maternal pregnancy outcomes. Our results demonstrated that intervention with dietary fiber supplement, from 20 to 24^+6^ gestational weeks in women with overweight/obesity prior to pregnancy, did lower the incidence of GDM, excessive weight gain, and preterm birth, but it did not confirm the positive effect of blood lipids.

Dietary fiber, the seventh most important dietary nutrient, plays a protective role in the improvement of glucose and lipid metabolism, weight control, and the regulation of intestinal flora during pregnancy (Zareei et al., 2018; Pretorius and Palmer, 2020; Tian et al., 2021). Compared to non-pregnant women, insulin sensitivity is decreased by approximately 50–60% in patients with GDM(Chiefari et al., 2017). Moreover, pregnant women with pre-pregnancy overweight/obesity have decreased insulin sensitivity as compared with lean or normal-weight women, which puts them at a higher risk of GDM(Johns et al., 2018). Dietary fiber has previously been associated with a reduced risk of GDM by several evidences (Goletzke et al., 2021; Pajunen et al., 2022). In our study, the incidence of GDM in women randomized to the fiber supplement group was significantly lower than in the control group. Consistent with the Australian longitudinal cohort study (3607 women; 12 years), women in the highest quartile of fiber intake had a 33% lower risk of GDM (*p* = 0.05) (Looman et al., 2018). Furthermore, Zhang et al. reported that women with the highest fiber intake in the first trimester or the second trimester, had an approximately 17%, or 18% lower risk of GDM, respectively (*p* ≤ 0.03) (Zhang et al., 2021).

An earlier meta-analysis demonstrated that fiber-rich diets benefit individuals with type 2 diabetes by lowering FPG and Hba1c levels (Post et al., 2012). Xie et al. observed a comparable impact of dietary fiber supplementation in improving glycemic control in type 2 diabetes, and they also found a convenient way to help individuals meet standard dietary fiber needs (Xie et al., 2021). After that, Cassidy et al. reported that dietary fiber, including β-glucan, inulin, guar gum, psyllium, resistant starch, and alginate, also had favorable effects on the regulation of postprandial plasma glucose (Cassidy et al., 2018). Our findings are consistent with prior research. After the intervention, women in the fiber supplement group had lower FPG and HbA1c levels than before the intervention. In addition, from the perspective of postprandial plasma glucose response, the value of the difference of 1 hPG and 2 hPG in the fiber supplement group was significantly higher than the control group, and the value of the difference of 2 hPG and FPG in the intervention group was significantly lower than the control group. These data may suggest that women demonstrated improved glucose tolerance following dietary fiber supplement. Moreover, a plethora of research has demonstrated that the mechanisms of glycemic control by fiber are strongly associated with the two predominant physicochemical properties of viscosity and fermentability (Gill et al., 2021; Malunga et al., 2021).

Lipid metabolism is essential for healthy pregnancy development, and women with GDM have increased concentrations of TG, TC, and LDL-C and lower levels of HDL-C (Wang et al., 2019). Wang et al. reported that the TG/HDL-c ratio could be a good marker to predict the risk of GDM(Wang et al., 2021). In addition, Observational studies have demonstrated that a daily fiber intake of 25 g or more can reduce the risk of cardiovascular disease by lowering blood lipids and cholesterol levels (Soliman, 2019; Kim et al., 2022). Similarly, Dehghan et al. conducted a randomized controlled clinical trial to determine the benefits of soluble fiber supplementation (10 g/day) on glycemic status and lipid profile in women with type 2 diabetes, and the results showed a significant reduction in FPG (8.50%), HbA1c (10.40%), TC (12.90%), TG (23.60%), LDL-C (35.30%), LDL-C/HDL-C ratio (16.25%),TC/HDL-C ratio (25.20%) and increased HDL-C (19.90%) (Dehghan et al., 2013). Discordantly, our study indicated that the TG and TG/HDL-C ratio levels in the fiber supplement group were statistically higher than those in the control group after the intervention, although the small differences were likely not clinically significant. A possible reason might be attributed to the blood lipids levels increases with the progression of gestational age mediated by human placental prolactin, and the dietary fiber might not be sufficient in inhibiting the physiological effects (Duttaroy and Basak, 2021). Furthermore, the phenomenon may also be owing to the limited sample size. This is a question that will need to be explored in future studies.

Gestational weight gain has been considered a potentially modifiable risk factor for GDM and other adverse pregnancy outcomes (Park et al., 2021). The Institute of Medicine (IOM) recommends that overweight/obese pregnant women gain less than 0.33 kg per week in the second and third trimesters (Khanolkar et al., 2020). Previous studies have demonstrated that increased dietary fiber intake contributes to satiety by increasing the volume of food in the stomach, hence reducing the calorie density of the meal and resulting in weight loss. (Atakan et al., 2021). Another study indicated that dietary fiber can be fermented by the gut bacteria into short-chain fatty acids (SCFAs), which stimulate the production of gut anorexic hormones such as glucagon-like peptide 1 (GLP-1) and peptide YY (PYY) from the L cells to reduce energy intake (Muller et al., 2018; Lin and Li, 2021). The study by Guess and others also shown that a high intake of dietary fiber had a positive impact on weight control in pre-diabetic patients (Guess et al., 2015). Consistently, we also found that higher fiber consumption was conducive to a significantly lower increase in total calories, carbohydrates, and protein intake in the fiber supplement group, suggesting that fiber might be beneficial for improving satiety, which may reduce food intake. This may be why women who were intervened by dietary fiber gained less weight than the control group. More importantly, the excessive weight gain went down by 21.3%, among women in the fiber supplement group.

Overweight/obesity prior to pregnancy was associated with an increased risk of unfavorable birth outcomes such as pregnancy-induced hypertension, preeclampsia, polyhydramnios, preterm birth (<37 weeks), cesarean section, and fetal macrosomia (Hashim et al., 2019; Ayensu et al., 2020; Liu et al., 2020). Adequate dietary fiber intake has potential health benefits for maternal and neonatal health outcomes. A prospective cohort research revealed that the VPR (vegetable, fruit, and white rice) dietary pattern, high in fiber, during pregnancy is related with a lower risk of preterm birth (OR: 0.55; 95% CI: 0.26–1.17, *p* < 0.01) (Chia et al., 2016; Zhang et al., 2017). Another meta-analysis of twenty-one studies found that adherence to a healthy dietary pattern (intake of vegetables, fruits, legumes, whole grains) was significantly associated with lower odds of preterm birth (OR: 0.75; 95% CI: 0.57–0.93, *p* = 0 < 0.01) and preeclampsia (OR: 0.78; 95% CI: 0.70–0.86; *p* = 0.178) (Kibret et al., 2018). Findings from the present study are consistent with those outlined above. Our study indicated that women in the intervention group had significantly lower rates of preterm birth compared with the control group. The significant improvements in pregnancy outcomes in the fiber supplement group might be related to the fact that 8.3% of these pregnancies were exposed to GDM compared with 24.0% in the control group. In addition, the lower rates of excessive weight gain may also have contributed. This will need to be explored in future meta-analyses with a larger set of studies.

Some limitations should be noted in our study. Firstly, the study was a unicentric, pilot study, and the sample size in the two groups was small, thus, the ability to assess accuracy may be limited. Secondly, estimation of dietary intake was self-reported by pregnant women, and subjective bias could be a concern. Finally, our intervention was only conducted from 20 to 24^+6^ weeks of gestation and did not continue to gestational age at delivery. Thus, we failed to demonstrate a positive effect of dietary fiber on the incidence of pregnancy-induced hypertension, preeclampsia, polyhydramnios, cesarean section, macrosomia, and neonatal distress respiratory syndrome.

## Conclusion

To sum up, our trial showed that supplement with dietary fiber during pregnancy among Chinese women who are overweight/obese prior to pregnancy was associated with a 15.7% decreased rate of GDM. In addition, dietary fiber also played a protective role in preventing excessive weight gain and preterm birth. However, we did not find its benefits in lowering blood lipids.

## Data Availability

The raw data supporting the conclusions of this article will be made available by the authors, without undue reservation.
