# Supplementary material for: The effect of dietary fiber supplement on prevention of gestational diabetes mellitus in women with pre-pregnancy overweight/obesity: A randomized controlled trial
Source: Front Pharmacol. 2022 Aug 29;13:922015. doi: 10.3389/fphar.2022.922015 (PMC9465204; doi:10.3389/fphar.2022.922015)
Supplement: Supplementary file 1 [file DataSheet1.docx]

Supplementary Material

# Supplementary Data

NO

# Supplementary Figures and Tables

**Table 1** Nutritional composition of soluble dietary fiber powder

| Project | Per 100 grams | NRV (%) |
| --- | --- | --- |
| Energy (kJ) | 904 | 11 |
| Protein (g) | 0 | 0 |
| Fat (g) | 0 | 0 |
| Carbohydrates(g) | 13.8 | 5 |
| Dietary fiber (g) | 81.5 | 326 |
| Sodium (mg) | 6 | 0 |

NRV: Nutrient Reference Values

**Table 2** Maternal descriptive characteristics at baseline

| Characteristic | Control  (n =50) | Intervention (n=48) | *P* |
| --- | --- | --- | --- |
| Gestational age (weeks) at  enrollment | 12.63±1.14 | 12.86±1.69 | 0.425 |
| Gestational age (weeks) at  OGTT | 25.58±1.20 | 25.57±0.75 | 0.948 |
| Age(years) | 29.96±4.07 | 31.13±4.21 | 0.167 |
| BMI (kg/m^2^) | 26.73±2.36 | 27.05±3.35 | 0.594 |
| Systolic blood pressure, (mmHg) | 116.60±12.76 | 119.42±11.93 | 0.262 |
| Diastolic blood pressure, (mmHg) | 71.68±9.11 | 72.19±10.03 | 0.794 |
| First pregnancy, n (%) | 20(40) | 11(22.9) | 0.069 |
| Family history of diabetes, n (%) | 8 (16) | 12(25) | 0.269 |
| PCOS, n (%) | 3 (6) | 7 (14.6) | 0.285 |
| Adverse pregnancy history, n (%) | 10 (20) | 6 (12.5) | 0.315 |

Data are expressed as mean ± SD or n (%). Control: standard prenatal care; Intervention: dietary fiber.

**Table3** Comparison of dietary intake within and between the group

| Variable | Control (n =50) | Intervention (n=48) | *P* |
| --- | --- | --- | --- |
| Calorie (kJ/day) |  |  |  |
| 20 weeks | 6747.36±1699.99 | 7041.85±1706.66 | 0.394 |
| 25 weeks | 7743.14±1248.14 | 7513.59±1437.79 | 0.400 |
| Energy from carbohydrate (%) |  |  |  |
| 20 weeks | 50.94±7.19 | 52.75±6.56 | 0.196 |
| 25 weeks | 47.90±4.51 | 47.35±4.60 | 0.554 |
| Energy from protein (%) |  |  |  |
| 20 weeks | 14.18±2.84 | 14.23±2.89 | 0.932 |
| 25 weeks | 17.70±2.10 | 17.81±2.71 | 0.818 |
| Energy from fat (%) |  |  |  |
| 20 weeks | 27.48±6.43 | 26.60±6.43 | 0.502 |
| 25 weeks | 28.26±4.01 | 26.96±4.59 | 0.138 |
| Dietary fiber (g) |  |  |  |
| 20 weeks | 15.00(12.63-18.94) | 15.37(12.18-18.005) | 0.693 |
| 25 weeks | 15.25(13.25-18.19) | 33.81(31.81-36.06) | <0.001 |

Data are expressed as mean ± SD or Median (IQR). Control: standard prenatal care; Intervention: dietary fiber.

**Table 4** Metabolism variables in pregnant women with overweight/obesity in the two groups before and after intervention

| Variable | Control (n =50) | | | *P* | Intervention (n=48) | | *P* |
| --- | --- | --- | --- | --- | --- | --- | --- |
|  | Baseline | End | |  | Baseline | End |  |
| HbA1c (%) | 5.16±0.37 | | 5.06± 0.37 | 0.033 | 5.18±031 | 5.00±0.33 | <0.001 |
| FBG (mmol/L) | 4.48±0.42 | | 4.37±0.58 | 0.150 | 4.57±0.38 | 4.41±0.29 | <0.001 |
| TC (mmol/L) | 4.76±0.77 | | 5.88± 0.81 | <0.001 | 4.78±0.96 | 5.82± 1.14 | <0.001 |
| TG (mmol/L) | 1.51±0.40 | | 2.19±0.54 | <0.001 | 2.27±1.18 | 2.70±0.82* | <0.001 |
| HDL-C (mmol/L) | 1.44±0.32 | | 1.81±0.31 | <0.001 | 1.46±0.36 | 1.74±0.38 | <0.001 |
| LDL-C (mmol/L) | 2.63±0.63 | | 3.19±0.81 | <0.001 | 2.64±0.86 | 3.19± 1.11 | <0.001 |

Data are expressed as mean ± SD. Control: standard prenatal care; Intervention: dietary fiber. Baseline: Gestational age (weeks) at enrollment; End: Gestational age (weeks) at OGTT. * Significantly different from control (*P* < 0.05).

**Table 5** Comparison of postprandial blood glucose between the two groups at the end of the intervention

| Blood glucose(mmol/L) | Control (n =50) | Intervention (n=48) | *P* |
| --- | --- | --- | --- |
| 1hBG | 7.74±2.13 | 7.81±1.19 | 0.850 |
| 2hBG | 6.83±1.83 | 6.31±1.27 | 0.094 |
| difference a | 3.38±1.92 | 3.39±1.11 | 0.955 |
| difference b | 0.91±1.18 | 1.49±1.54 | 0.037 |
| difference c | 2.47±1.60 | 1.90±1.07 | 0.042 |

Data are expressed as mean ± SD. Control: standard prenatal care; Intervention: dietary fiber. 1hBG: 1-hour postprandial blood glucose; 2hBG: 2-hour postprandial blood glucose; Difference a:1hBG-BPG, Difference b: 1hBG-2hBG, Difference c: 2hBG-FPG

**Table 6** Comparison of body weight before and after intervention in the two groups

| Weight(kg) | Control (n =50) | Intervention (n=48) | *P* |
| --- | --- | --- | --- |
| W20 | 72.20±8.54 | 71.66±6.97 | 0.733 |
| W25 | 74.73±8.673 | 73.65±6.92 | 0.498 |
| W25 - 20 | 2.53±1.20 | 1.99±1.09 | 0.022 |

Data are expressed as mean ± SD. Control: standard prenatal care; Intervention: dietary fiber. W20: maternal weight at 20 weeks of gestation, W25: maternal weight at 25 weeks gestation, W25-20: maternal weight increased between 20 and 25 weeks of gestation.

## Supplementary Figures

**Figure 1 Clinical trial flow diagram.**

**
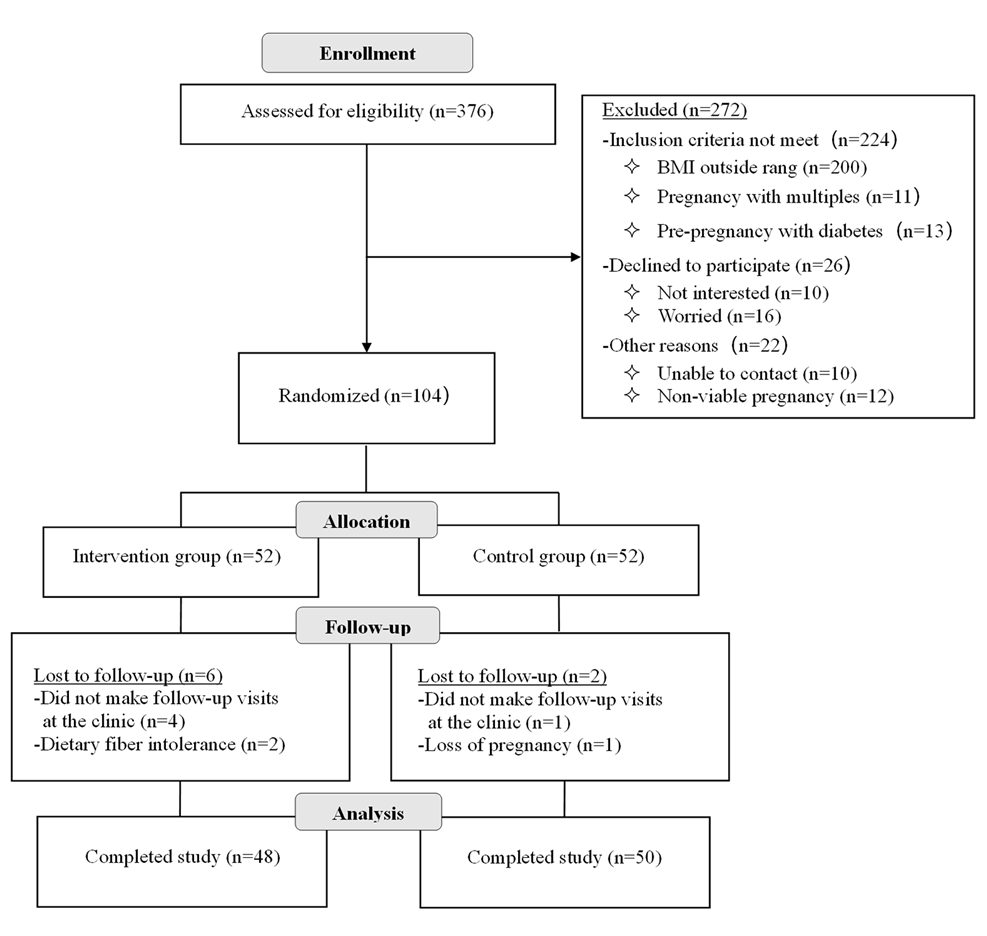
**
